# Molecular profiling for precision cancer therapies

**DOI:** 10.1186/s13073-019-0703-1

**Published:** 2020-01-14

**Authors:** Eoghan R. Malone, Marc Oliva, Peter J. B. Sabatini, Tracy L. Stockley, Lillian L. Siu

**Affiliations:** 1 Division of Medical Oncology and Hematology, Princess Margaret Cancer Centre, University Health Network, Department of Medicine, University Avenue, University of Toronto, Toronto, Ontario M5G 1Z5 Canada; 2 Department of Clinical Laboratory Genetics, University Health Network, and Department of Laboratory Medicine and Pathobiology, University of Toronto, Toronto, Canada

## Abstract

The number of druggable tumor-specific molecular aberrations has grown substantially in the past decade, with a significant survival benefit obtained from biomarker matching therapies in several cancer types. Molecular pathology has therefore become fundamental not only to inform on tumor diagnosis and prognosis but also to drive therapeutic decisions in daily practice. The introduction of next-generation sequencing technologies and the rising number of large-scale tumor molecular profiling programs across institutions worldwide have revolutionized the field of precision oncology. As comprehensive genomic analyses become increasingly available in both clinical and research settings, healthcare professionals are faced with the complex tasks of result interpretation and translation. This review summarizes the current and upcoming approaches to implement precision cancer medicine, highlighting the challenges and potential solutions to facilitate the interpretation and to maximize the clinical utility of molecular profiling results. We describe novel molecular characterization strategies beyond tumor DNA sequencing, such as transcriptomics, immunophenotyping, epigenetic profiling, and single-cell analyses. We also review current and potential applications of liquid biopsies to evaluate blood-based biomarkers, such as circulating tumor cells and circulating nucleic acids. Last, lessons learned from the existing limitations of genotype-derived therapies provide insights into ways to expand precision medicine beyond genomics.

## Background

**Fig. 1:**
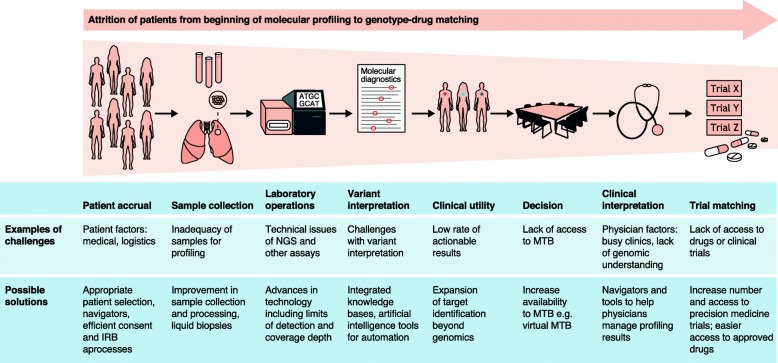
The process from genetic sequencing of patients to enrollment on genotype-matched clinical trials. MTB, molecular tumor board; IRB, institutional review board; NGS, next-generation sequencing

In the past decade, the field of oncology has witnessed substantial changes in the way patients with cancer are managed, with departure from a “one-size-fits-all” approach and increasing focus on precision medicine based on genomic variants. Cancer precision medicine is defined as “the use of therapeutics that are expected to confer benefit to a subset of patients whose cancer displays specific molecular or cellular features (most commonly genomic changes and changes in gene or protein expression patterns)” [1]. In certain tumors, molecular profiling may also yield clinically relevant diagnostic and prognostic information. Owing to the genomic complexity of cancers, precision medicine has been enabled by a growing body of knowledge that identifies key drivers of oncogenesis, coupled with advances in tumor analysis by next-generation sequencing (NGS) and other profiling technologies, and by the availability of new therapeutic agents. Precision medicine has already transformed cancer care: both common and rare malignancies can be targeted by specific therapies to improve clinical outcomes in patients (Table 1). This review focuses on current and emerging approaches, highlights successes and challenges, and proposes potential solutions in the implementation of precision medicine in clinical research and practice (Fig. 1). The expansion to other molecular characterization technologies beyond genomics, such as transcriptomics, epigenetics, and immunophenotyping, and to the evaluation of drug combinations beyond monotherapy approaches will hopefully increase the clinical utility and scope of precision medicine. Last, patients represent active key stakeholders in precision medicine initiatives; thus, resources must be deployed to optimize their education and engagement.

**Table 1:** FDA and EMA approved biomarker matching targeted drugs and routine molecular pathology testing [2, 3]

Gene/protein	Anticancer agent	Indications	Biomarker	Routine testing
*ALK*	Crizotinib, ceritinib, alectinib, lorlatinib, brigatinib	NSCLC	*ALK* translocation	FISH, IHC
Androgen receptor (AR)	Abiraterone, enzalutamide, dalurotamide, apalutamide	Prostate cancer	AR expression	IHC
*BCL-2*	Venetoclax	Chronic myeloid leukemia	BCL-2 protein expression, *BCL-2* amplification/translocation	IHC, FISH
*BCR/ABL*	Imatinib, dasatinib, nilotinib, bosutinib, ponatinib	Chronic myeloid leukemia	*BCR/ABL1* fusion	IHC (FISH, DNA/RNA sequencing), PCR^1^
*BRAF*	Dabrafenib+trametinib, vemurafenib+cobimetinib, encorafenib+binimetinib	Melanoma, NSCLC, anaplastic thyroid cancer, hairy cell leukemia	*BRAF* V600E/K mutations	IHC, PCR^1^, DNA sequencing
*BRCA*	Olaparib, talazoparib, rucaparib	Breast cancer, ovarian cancer	Germline/somatic *BRCA* 1/2 mutations	DNA sequencing
*C-KIT*, *PDGFR*	Imatinib	Gastrointestinal stromal tumor	*c-KIT* Exon 9 and 11 mutations, *PDGFR* mutations	IHC, DNA sequencing
*PDGFRB*	Imatinib	Myelodysplastic/myeloproliferative syndromes	*PDGFRB* rearrangement	FISH
Estrogen/progesterone receptors (ER/PR)	Tamoxifen, raloxifene, fulvestrant, toremifine	Breast cancer	ER/PR expression	IHC
*erBB2/HER-2*	Trastuzumab, pertuzumab, ado-trastuzumab, emtansine, neratinib	Breast cancer, gastric cancer	HER-2 protein expression, *HER-2* amplification	IHC, FISH
*EGFR*	Gefitinib, erlotinib, afatinib, dacomitinib	NSCLC	*EGFR* exon 19 deletion, exon 21 L858R mutation	DNA sequencing, PCR^1^
	Osimertinib		*EGFR* T790M mutation	
*FGFR2/3*	Erdafitinib	Bladder cancer	*FGFR3* mutations, *FGFR2/3* fusions	DNA sequencing, FISH
*FLT3*	Midostaurin, gilteritinib	Acute myeloid leukemia	*FLT3* mutations	DNA sequencing, PCR^1^
*IDH1/2*	Ivosidenib, enasidenib	Acute myeloid leukemia	*IDH1/2* mutations	IHC, DNA sequencing
*MET*	Crizotinib (breakthrough designation)	NSCLC	*MET* amplification, *MET* exon 14 alterations	FISH, DNA/RNA sequencing
MSI-H or dMMR	Pembrolizumab	MSI-H or dMMR solid tumors	MLH1, MSH2, MSH6, PMS2 protein expression, MSI high	IHC, DNA sequencing, PCR^1^
	Nivolumab and ipilimumab	Colorectal cancer		
*NTRK*	Larotrectinib, entrectinib	Solid tumors with NTRK fusions	*NTRK* protein expression, *NTRK* fusion	IHC, FISH, DNA/RNA sequencing
*PI3KCA*	Alpelisib	Breast cancer	*PI3KCA* mutation	DNA sequencing
*PI3K* (alpha and delta)	Copanlisib	Follicular lymphoma	*PI3K* mutation	DNA sequencing
*PI3K* (delta and gamma)	Duvelisib	Chronic lymphocytic leukemia, small lymphocytic lymphoma	*PI3K* mutation	DNA sequencing
*RAS* (negative predictor)	Cetuximab, panitumumab	Colorectal cancer	*KRAS/NRAS* wildtype	DNA sequencing
*RET*	LOXO-292 (breakthrough designation)	NSCLC, medullary thyroid cancer	*RET* fusion, *RET* mutation	FISH, DNA/RNA sequencing
*ROS1*	Crizotinib, entrectinib	NSCLC	*ROS* translocation	FISH, DNA/RNA sequencing

*AR* androgen receptor, *dMMR* deficient mismatch repair, *ER* estrogen receptor, *FISH* fluorescence in situ hybridization, *IHC* immunohistochemistry, *MSI-H* high levels of microsatellite instability, *NSCLC* non-small cell lung cancer, *PR* progesterone receptor

^1^Applications of PCR may include fragment analysis, quantitative PCR, and restriction fragment length polymorphisms

## Current and emerging molecular approaches to enable precision medicine

### Integration of precision medicine with other diagnostic tests in clinical practice

First and foremost, the important interactions between research and standard of care testing in precision cancer medicine must be highlighted. Large-scale research studies may identify new biomarkers with clinical utility, which can then be taken up as a new standard of care clinical diagnostic test to maximize the benefit to the patient population. Many tumor-specific molecular alterations, including protein overexpression, mutations in driver genes, or rearrangements, are well-proven predictive biomarkers of response to selective targeted therapies, with additional biomarkers rapidly emerging. Clinical molecular pathology analysis has therefore become an indispensable laboratory tool that can be used to characterize tumor biology and to drive therapeutic decisions.

Conventional tests such as immunohistochemistry (IHC) and fluorescence in situ hybridization (FISH) are fundamental precision medicine tools in daily practice [4], with many biomarkers currently detected by these two techniques (Table 1). IHC can detect changes at the protein level that result not only from gene aberrations, most commonly gene amplifications, but also from specific DNA rearrangements or point mutations (such as *EML4-ALK* translocation in non-small cell lung cancer (NSCLC) and *IDH1/2* mutations in glioma) [5–7]. The use of IHC has extended to biomarkers of response to immuno-oncology (IO) agents, including PD-L1 expression or mismatch repair status, which determine the eligibility for therapies that are based on anti-PD-1/PD-L1 agents in certain tumor types [1, 4, 5, 8–10]. FISH has been the gold-standard technique for determining DNA rearrangements, and it is also routinely used to confirm amplifications at the gene level when IHC results are equivocal [11, 12]*.*

As the number of druggable gene aberrations and predictive biomarkers grow in oncology, NGS technologies have increasingly substituted for conventional techniques, such as single-gene testing, and for targeted mutational platforms in routine molecular pathology. Conventional techniques have high sensitivity and specificity in detecting actionable mutations with proven benefit from matched targeted therapies or in identifying mutations that are associated with resistance to specific drugs [13–16]. However, with these techniques, each biomarker must be pre-specified in order to be detected and a purpose-made assay is required for each analyte. NGS can enable the simultaneous analysis of a broad spectrum of genomic alterations, including mutations, copy number variations (CNV), translocations, and fusions in multiple genes. It therefore provides a more efficient, cost- and tissue-saving tumor analysis as compared to serial single-biomarker analyses [17, 18], particularly in the context of the genomic complexity that is known to exist within tumors. Several studies comparing NGS performance against reverse transcriptase (RT)-PCR-based targeted mutation platforms, single-gene testing and other conventional techniques have shown similar sensitivity and specificity in detecting common druggable genomic aberrations in daily practice [19–21]. Given the decreasing costs and turnaround time of NGS, the improvement in bioinformatics analyses, and the harmonization of knowledgebases to facilitate the clinical interpretation of genomic results, the move to comprehensive genomic profiling by NGS in clinical testing is compelling in the precision cancer medicine context.

### Current applications of NGS approaches: targeted gene panels vs whole exome and whole genome sequencing

NGS can be limited to a pre-specified group of genes (targeted gene panels), can focus on the coding regions of all of the base pairs of the genome (whole exome sequencing (WES)), or can involve the analysis of the entire tumor genome, including the intronic regions (whole genome sequencing (WGS)). The choice between these approaches depends on several factors, including the final application of tumor testing (clinical vs research), the results required, technical efficiency, and cost (Additional file 1) [17]. To date, WES and WGS have been largely confined to the research space, with the goal of collecting large amounts of genomic information for translational research applications that can improve knowledge in cancer biology over time. Targeted gene panels have been used preferably in the clinical setting because they provide greater depth of coverage in selected areas of interest (e.g., hotspot regions with known actionable mutations), faster turnaround, and more clinically relevant data when compared to broader genomic profiling by WES or WGS approaches [22]*.* The number of genes included in these panels can vary, ranging from 20–30 to over 400–500 genes. Although the clinical utility of assessing all of the genes that are included in large panels is currently uncertain, the benefit of simultaneous multi-gene testing using NGS and the low incremental cost of including additional genes are motivators for using more comprehensive genomic profiling in the clinic.

A significant challenge is that although large-scale tumor sequencing studies and genotype-matched trials have reported actionable mutations in driver genes in up to 40% of the patients, a significantly lower proportion of patients (10–15%) end up being treated with genotype-matched drugs [23–28]. Multiple factors are at play, but the key challenge is the lack of approved or investigational agents to match specific driver alterations. In cases where the target molecular aberration occurs infrequently, the recruitment of patients who have such rare mutations into clinical trials can be challenging and can hinder the development of new drugs. Furthermore, intratumoral heterogeneity (e.g., trunk vs branch mutations) and whether or not a specific molecular alteration is a “true” driver in a particular tumor can ultimately impact the efficacy of the matched therapy [29]. In this regard, Hess et al. [30] have recently revealed that many somatic hotspot mutations that are thought to be involved in tumorigenesis and labeled as “drivers” might actually be recurrent passenger mutations, which occur in areas of the genome that are highly mutable.

A recent retrospective analysis in NSCLC showed no survival gain in patients who underwent genomic profiling using multi-gene targeted panels compared to patients who underwent only single-gene testing of *EGFR* and *ALK* genes, with panel testing offering additional opportunities for targeted therapy to fewer than 5% of patients [31]. However, broader genomic analyses, looking beyond actionable variants in known genes, have the potential to inform on acquired resistance to treatments (for example, the *EGFR T790M* mutation confers resistance to first-generation EGFR tyrosine kinase inhibitors (TKI) in NSCLC) or to suggest potential synergistic drug combinations (for example, downstream activation of the BRAF pathway led to the combination of BRAF and MEK inhibitors in *BRAF* mutant melanoma) [32, 33]. The abovementioned findings resulted in the incorporation of new treatment options in patients with *EGFR*-mutant NSCLC (such as osimertinib) [34, 35] and *BRAF*-mutant NSCLC [36, 37] and melanoma (such as the dabrafenib and trametinib combination) [38], which have led to a significant improvement in survival in these patient populations and ultimately changed the standard of care.

Advances in sequencing technologies such as WGS have facilitated the simultaneous detection of single nucleotide variants (SNV), CNV, and structural rearrangements such as gene fusions, leading to greater diagnostic yield of actionable findings in tumor samples. The value of performing comprehensive genomic profiling is exhibited by a recent study that characterized 2520 samples of metastatic tumors with paired normal tissue. WGS was used to identify the catalog of genetic mutations found in each metastasis, thus illuminating the genomic changes present in the metastases of 22 solid tumors, and 62% were found to contain at least 1 actionable event [39]. Similar evaluations were also performed on pediatric pan-cancer cohorts to identify driver genes [40]. New sequencing approaches have extended the length of sequencing fragments to more than a few kilobases, thus improving the ability to detect complex structural alterations in the genome [41]. One study using genomic DNA from patients who had a variety of brain cancers was able to detect SNV, CNV, and methylation profiles simultaneously from a low-pass WGS approach using long-read sequencing [42]. In cancer, most long-read sequencing efforts have focused on RNA sequencing and have discovered novel fusion and splicing isoforms that are relevant to tumor progression or treatment resistance [43–46]. Currently, the error rates for long-read technologies are too high for somatic variant detection, but the movement towards these approaches for tumor analysis would provide a holistic approach to genomic tumor profiling and would improve personalized therapeutic management.

### Circulating biomarkers

The quality, quantity, and availability of tumor tissue from cancer patients pose challenges to the clinical implementation of precision medicine. The processing of formalin-fixed, paraffin-embedded fragments can alter nucleic acids, and low tumor content in tumor samples can decrease test sensitivity or lead to false-positive mutation calls [17]. In addition, the use of archival tissue or biopsies collected at a single time point may not account for intratumoral heterogeneity in space or time [47–49]. Acquisition of multiple tumor biopsies to overcome this is hindered by the need for invasive procedures that not only put patient safety at risk but also require a significant amount of resources.

An emerging field that may ameliorate some tumor sample issues is the testing of circulating tumor-specific markers. These include circulating tumor cells (CTC) or circulating tumor DNA (ctDNA), as well as RNAs, proteins, or metabolites, that are present in body fluids such as the blood, urine, and peritoneal or cerebrospinal fluid [50–54]. Liquid biopsies are easily accessible through minimally invasive procedures that can be repeated to provide a dynamic and longitudinal assessment of tumor-specific diagnostic, prognostic, or predictive biomarkers. NGS can be applied to plasma CTC and ctDNA, providing a more comprehensive picture of the tumor genomic landscape than can be obtained from tumor tissue, as it reflects different tumor regions (such as primary and metastatic sites) and thus tackles intratumoral heterogeneity [55, 56]. Single-cell analysis of CTC also allows multi-omic assessment and enables the generation of patient-specific tumor models, such as organoids and xenografts. Viable CTC are thought to be involved in the formation of tumor metastasis and thus may reflect a metastatic genotype. Therefore, single-cell analyses can lead to the detection of actionable aberrations that are implicated in metastatic spread [57], whereas tumor models derived from CTC might serve to test novel drugs or sensitivity to current standard therapies [58, 59].

Potential clinical uses of blood-based CTC and ctDNA that are not offered by tumor tissue sequencing include monitoring the persistence of radiologically undetectable tumors (e.g., minimal or molecular residual disease), prediction of recurrence (e.g., persistent CTC associated with risk of relapse in breast cancer) [60], monitoring of treatment response (e.g., CTC dynamics in prostate and breast cancer patients treated with chemotherapy) [61, 62], early detection of resistance mechanisms [63, 64], assessment of tumor burden (e.g., correlation between tumor burden and ctDNA variant allele frequency in NSCLC) [65], tracking the clonal evolution of tumors [66], and dynamic evaluation of immune biomarkers such as PD-L1 expression and tumor mutation burden [67–69].

To date, two tests based on plasma ctDNA analysis have been FDA-approved for clinical use: the testing of *EGFR* mutations in patients with NSCLC and the methylation of the *SEPT9* promoter as screening for colorectal cancer [56, 64, 67, 70]*.* Topics that remain to be addressed include the high variability of CTC and plasma ctDNA levels between individual patients that result from inconsistent shedding and the impact of disease burden and/or cancer type on circulating nucleic acids [47].

Beyond CTC and ctDNA, other circulating tumor biomarkers such as RNA, proteins, and metabolites are still in early phases of development and need to be explored properly. At present, there are no FDA-approved assays for the detection and analysis of these biomarkers and their clinical utility remains unclear [50]. Circulating-free microRNAs (miRNA) are of particular interest because of their stability and high abundance in the plasma, and miRNA signatures are being investigated as diagnostic and prognostic biomarkers in several tumor types, including for the detection of minimal residual disease [71–73]. However, the variability and lack of reproducibility of the results across studies, which result from the lack of standardized methods for collection and analyses, remain the main challenges [74, 75]. There is an urgent need for methodological standardization to exploit the full potential of liquid biopsies in the clinic, and multiple initiatives to address this need are ongoing [75].

## Taking actions on genomic results

### Identification and clinical interpretation of genomic results

The interpretation of the clinical impact of tumor variants in the context of a specific cancer and for individual patients is an active field of study in precision cancer medicine [63]. To facilitate variant annotation and clinical interpretation, genomic databases and online resources have compiled associations with a specific histology or disease, as well as their prognostic and/or predictive value of response to specific therapies [76]. The data collected in these knowledgebases vary depending on their final scope, ranging from pre-clinical biological and functional data for translational research applications (e.g., The Cancer Genome Atlas (TCGA), International Cancer Genome Consortium (ICGC)) to the most updated evidence on the clinical benefit obtained from matched drugs that can be used to drive variant-specific treatment recommendations (e.g., OncoKB, MyCancerGenome, CIViC) [77, 78]. Other sources are specifically focused on passenger variant curation (e.g., dbCPM) [79]. Datasets such as MyCancerGenome or CIViC also help to discriminate driver variants (which are implicated in tumor growth and proliferation) from passenger tumor variants (incidental variants that do not confer survival or clonal advantage). Of note, these datasets might need to be revised in the light of the recent findings from Hess et al. [30] (see the “Current applications of NGS approaches: targeted gene panels vs whole exome and whole genome sequencing” section). Current statistical models that are used to account for background tumor mutability at the gene level, in an attempt to discriminate between driver and passenger mutations, are inaccurate and might lead to high rates of false positives, i.e., identification of driver variants that are actually passengers. The new model proposed by Hess et al. [30] accounts for mutability at the level of individual base pairs and, as such, has increased power and specificity to distinguish true driver mutations. In those cases where annotated variants are unknown or have not been previously reported, a few available website-based approaches can provide information on the predicted biological effects of novel variants on the basis of in silico tools and meta-prediction methods (e.g., dbNSFP) [80, 81]. Germline-based genomic databases (e.g., *National Center for Biotechnology Information* (NCBI) ClinVar, *Online Mendelian Inheritance in Man* (OMIM)) that compile previously reported germline polymorphisms may also aid in differentiating tumor-specific somatic variants from non-pathogenic DNA variations in patients for whom no available matched normal tissue or blood is available [82, 83].

One of the major downsides of having multiple knowledgebases is the dispersion of the genomic information. New user-friendly tools have been developed to integrate the knowledge from established databases on diseases, genes, variants, and drugs (from basic biology to clinical evidence) in a single space, with the ultimate goal of improving clinical interpretation by healthcare professionals [84]. In addition, the existence of independent resources with different curators and scopes can lead to inconsistent or incomplete data collection on variants across platforms and to different format presentation and nomenclature, which can generate knowledge gaps and thus hamper the interpretation of variant actionability. Continued efforts to standardize variant curation and cataloging are needed to guarantee the utility of the genomic data provided. In this regard, a consensus set of minimal variant level data (MVLD) for tumor variant curation with a focus on clinical utility has been proposed by the Somatic Working Group of the Clinical Genome [84]. The generated data framework goes beyond genomic descriptive information to include data on clinical impact, such as biomarker class (diagnostic, prognostic, predictive), matched drugs available, and therapeutic effect (responsive vs resistant)*.* Other global harmonization initiatives for variant curation and interpretation, such as the Global Alliance for Genomics Health (GA4GH) Variant Interpretation for Cancer Consortium (VICC), have also been proposed [85].

The implementation of guidelines and consensus to standardize somatic variant annotation, classification, and reporting is critical to enable the interpretation of variants across institutions and professionals. The Association for Molecular Pathology, American Society of Clinical Oncology, and College of American Pathologists have published a series of recommendations for the classification and reporting of somatic variants in cancer patients, which are based on their clinical significance and the available supporting evidence [86]. Evidence-based variant categorization aims to help clinicians in translating the potential actionability of somatic variants into clinical decision-making.

The detection of clinically relevant germline mutations in patients undergoing tumor genomic profiling has been reported [86, 87]. This is especially important for cancers with a large inherited component, such as breast, ovarian, and colorectal cancers. For example, the molecular assessment of colorectal cancer to identify sporadic vs inherited Lynch syndrome has traditionally involved a multi-step approach, which uses sequential testing mismatch repair proteins by IHC, microsatellite instability, and then additional molecular testing for somatic changes to rule out sporadic cases. Upfront tumor profiling with an NGS panel that includes sequencing for mismatch repair proteins, sequencing for other recurrent somatic changes (e.g., *BRAF*), and assessment of microsatellite instability proved to have greater sensitivity than IHC in identifying Lynch syndrome in patients with colorectal cancer [88]. For ovarian cancer, somatic tumor profiling of *BRCA1* and *BRCA2* for PARP inhibitor therapy may reveal inherited germline mutations in these genes. To address these issues, recent guidance from the European Society of Medical Oncology Precision Medicine Working Group recommends that germline-focused analysis should be performed during tumor-only genomic profiling to identify variants with high allele frequencies (> 20–30%) and selected genes of clinical relevance [87]. Referral to genetic subspecialties is also recommended for familial management and long-term follow-up.

### Molecular tumor board

Large-scale genomic sequencing is currently available through academic institutions and private enterprises and is now funded in some jurisdictions but not in others; for instance, funding is now provided by Medicare in the USA. Target–drug matching can become increasingly complex as more information becomes available through the use of large panel tests or WES/WGS approaches. There are expanding numbers of patients with complex genomic data that are in need of interpretation. In order to exploit the potential of NGS-driven therapy fully, a formal entity such as a molecular tumor board (MTB) should exist that brings interdisciplinary expertise into the evaluation of patients who have advanced cancer to indicate when alteration-driven treatment is advisable. These multi-disciplinary teams typically include oncologists, research scientists, bioinformaticians, pathologists, medical geneticists, genetic counselors, and genomicists, among others. They examine each patient’s clinical, pathologic, and molecular information, review the literature and available resources, carry out discussions to reach a consensus if possible, and make treatment suggestions [89]. Previous studies have shown that interdisciplinary tumor boards can result in significant changes in treatment decisions [90–93]. The impact of MTB on outcomes has not yet been studied in-depth, but they can help to identify patients for clinical trials, educate participants, facilitate collaboration, and ensure that providers across multiple locations are testing and treating patients in a uniform and consistent manner, based on clinical practice guidelines and best available evidence.

Published studies have identified a knowledge gap and lack of confidence of physicians in their ability to interpret sequencing data. For instance, 22% of physicians at a tertiary cancer center reported a lack of confidence in their genomic knowledge, so there is clearly a need to educate oncologists in interpreting genomics data [94]. Younger oncologists have been found to be more likely to use NGS testing than older colleagues. Physicians who have access to an MTB have also been found to increase their use of NGS [95]. MTB can improve clinicians’ understanding of assay strengths, limitations, and results; can increase oncologists’ confidence in the application of molecular diagnostics; and ultimately can enhance the success of precision medicine.

There are various challenges to implementing a successful MTB; for instance, it is not always possible for members to meet in person, the MTB may not always be accessible by community oncologists, and there is a lack of standard quality requirements and guidelines on how to run an MTB and make treatment decisions [90]. A solution to some of these issues is the use of virtual MTB. Interactive virtual MTB allows participation by a variety of healthcare professionals across a wide geographic area. In addition, virtual MTB can involve both a major academic center and a community cancer program to facilitate information exchange and maximize clinical trial accrual. The development of guidelines can be achieved by deriving broad-based consensus from experts in MTB panels and those in professional associations.

### Applicability of genomic results outside of approved indications

The application of NGS may provide the treating physician with a list of druggable alterations. However, approved drugs are often inaccessible to biomarker-positive patients who have different tumor types because of a lack of reimbursement for drugs that are being used beyond their labeled indications. As a result, patients either need to be treated within the auspice of clinical trials or enrolled in compassionate access programs. Most clinical trials only cover a minority of potential genomic treatment indications and often have strict inclusion and exclusion criteria. Some molecular screening programs have performed gene panels to identify patients for opportunistic enrollment into early phase trials of targeted agents, whereas others have channeled patients to prospective biomarker-driven studies that sought out specific aberrations [24, 25, 96–98].

Large-scale tumor profiling studies using NGS have revealed significant genomic similarities, with shared actionable alterations in driver genes, among different tumor types (e.g., *BRAF* mutations are found across multiple tumor types) [99, 100]*.* As a result, the paradigm of precision oncology has shifted to “pan-cancer” biomarker-based approaches for therapeutic selection. The predictive value of *NTRK* fusions as biomarkers of response to TRK kinase inhibitors (larotrectinib, entrectinib) is a successful illustration of this approach. Both drugs have now been approved by the FDA to treat all solid tumors carrying *NTRK* fusions, and thus, TRK kinase inhibitors represent the second approved group of tissue-agnostic drugs in cancer, following pembrolizumab for patients with MSI-high tumors [101]. Nevertheless, the implementation of pan-cancer biomarker testing in routine practice is challenging. The incidence of actionable genome aberrations is low overall and highly variable across tumor types, necessitating the testing of large numbers of tumors with significant use of resources. In the specific case of *NTRK* fusions, a diagnostic algorithm based on the incidence by tumor type and NTRK expression by IHC testing has been proposed as a more efficient detection strategy in routine practice [102–105].

## Ways to expand precision medicine

### Mutational signatures

As discussed, genomic profiling for cancer precision medicine has a significant focus on finding discreet driver mutations that are associated with therapeutic targets or that are of diagnostic or prognostic value. An additional genomic tool in cancer are genomic “profiles” that harbor similar patterns of gene expression or of inherited or somatic mutations across multiple genes or genomic regions. With proper analysis, it is possible to group patients into subcategories for response, outcomes, or other clinical features. Mutational signatures expand genomics beyond the simple focus of discreet variant detection, with risk profiles reported in numerous cancer types including hepatocellular carcinoma, breast cancer, brain cancer, and diffuse large B cell lymphoma [106–108]. These approaches offer the potential for increased diagnostic yield, as a conventional single gene or panel testing cannot account for the complete array of mutational impacts. However, one study found that germline mutations in *BRCA1* and *BRCA2* responded to carboplatin, whereas those with a *BRCA* mutational signature and no germline variant did not respond [109]. More clinical evaluations are needed to understand the impact of mutational signatures and response to therapeutic targets.

### Gene expression signatures

The most advanced use of gene signatures is gene expression profiling from RNA sequencing (RNAseq), gene expression microarrays, or other single-molecule enumeration methods that are used to subclassify tumors into gene expression signatures. For example, gene expression arrays are used to provide consensus molecular subtyping of colorectal cancer [110]. Mutated signatures that suggest “BRCAness” in breast, ovarian, and prostate cancers predict response to PARP inhibitors [111, 112]. Single-molecule enumeration technologies can generate gene expression counts and have been used in many disease sites to characterize expression signatures. Examples include additional subgroups of diffuse large B cell lymphoma and also a prognostic prediction of disease recurrence in breast cancer [113, 114]*.* Numerous other breast cancer recurrence risk testing platforms that use expression signatures are also available and incorporated into clinical practice guidelines [115]. These studies highlight the improved clinical sensitivity of gene expression signatures relative to single gene mutation testing, as many mutated signature profiles did not have a canonical mutation found in the respective gene. The detection of gene expression networks and of the activity of oncogenic pathways through transcriptomic analyses can add a more “functional” tumor profiling that can ultimately increase treatment opportunities [116]. The Worldwide Innovative Network (WIN) Consortium recently evaluated the feasibility and clinical utility of adding transcriptomic analysis to tumor genotyping (WINTHER study) [117]. In this study, patients were first evaluated for targetable alterations in cancer driver genes; if none were present, the patients received treatment tailored to differences in gene expression between the patients’ tumor and normal tissue. The study showed that the addition of transcriptomic analysis to genomics increased actionability, with 35% of patients receiving matched targeted therapies. Overall, the efficacies of transcriptome-matched drugs appeared similar when compared with those of genotype-matched drugs, with responses ranging between 20 and 30% [117]. A similar study led by the German Consortium Group is now ongoing and may add more information in this regard; the workflow involves NGS and other omics technology, bioinformatics processing, validation of variants, and clinical evaluation at MTB to match patients to treatment [118].

### Role of epigenetics in precision medicine

Epigenetic changes modify the genome in order to modulate transcriptional activity that ultimately generates a permissive or restrictive architecture for cell growth and proliferation [119]. The epigenetic changes include the methylation of CpG islands in promoter regions, histone acetylation, and the association of non-coding RNA molecules (e.g., microRNA) with promoter regions. These epigenetic modifications can be detected using numerous technologies, including bisulfite sequencing, methylation microarrays, and chromatin immunoprecipitation sequencing arrays. Although many oncogenic targets of epigenetic pathways still rely on the detection of classic mutations found in genes that are involved in epigenetic modifications, such as *DNMT* and *EZH2*, genome-wide epigenetic maps of DNA methylation and histone modifications are being developed (e.g., International Human Epigenetic Consortium or NIH roadmap Epigenomics Mapping Consortium) [120, 121]. These epigenetic mapping efforts aim to help to granulate tumor biology and therapeutic potential for clinical action. Emerging data describing the role of epigenetic changes in oncogenesis and cancer progression pave the way for early therapeutic intervention or pharmacological targeting. For example, in pre-invasive lung cancer lesions, DNA methylation profiles are distinct between progressors and regressors [122]. Simultaneous mutations in *IDH2* and *SRSF2* genes promote leukemogenesis through coordinated effects on the epigenome and RNA splicing [123]. Genome-scale DNA methylation mapping demonstrates heterogeneity in time and space between primary and recurrent glioblastoma [124]. High and low CpG island methylator phenotypes in colorectal cancer are associated with *BRAF* mutations or *KRAS* mutations, respectively [125]. Although epigenetic targeting as a precision medicine strategy is complex and requires prospective clinical evaluation, the accumulating knowledge in this area will increase its therapeutic potential over time.

### Integration of PCM in the IO era

Beyond the protein expression of immune checkpoint molecules such as PD-L1, genomic analyses also play a role in predicting response or resistance to IO agents [126]. Tumor mutation burden (TMB), defined as the total number of coding mutations in the tumor genome, has emerged as a promising predictive biomarker of response to anti-PD-1/PD-L1 agents in several prospective trials, which have included multiple tumor types [127–129]. TMB can be assessed either on tumor samples or using ctDNA from blood samples [130, 131]. However, the cutoff values and the size and content of the genomic footprint required for TMB analysis are still not clear [132], and harmonization initiatives are underway to standardize the approach to interpreting tumor mutation for therapeutic uses (e.g., Friends of Cancer TMB initiative Quality Assurance Initiative Pathology) [133]. TMB is not predictive of response to anti-PD-1/PD-L1 agents across all cancers, as a few tumor types, such as Merkel cell carcinomas, are quite responsive to IO agents despite having a relatively low TMB [134, 135]. The presence of genomic aberrations affecting specific immune signaling pathways or genes that will ultimately lead to immune dysregulation (e.g., loss-of-function mutations in beta-2 microglobulin (B2M) or human leukocyte antigen (HLA) genes, *PTEN* loss, or mutations in *JAK* or other IFNγ-related genes) can be informative of resistance to immune checkpoint inhibitors [126, 136–138]. In addition to genomics, transcriptomic analyses can be used to define gene expression profile signatures that can be used to identify tumors that are more likely to respond to IO agents. For example, a “T cell-inflamed” gene expression profile was recently shown to be predictive of response to anti-PD-1/PD-L1 agents, regardless of tumor type [127].

### Evolving scope of precision cancer medicine

The field of precision oncology is moving from isolated genomic analyses towards a multi-omic approach to achieve a better understanding of tumor biology and to increase treatment opportunities. The ACNS02B3 brain tumor biology study, led by the Children’s Oncology Group across several institutions, represents a successful example of expanding molecular profiling beyond genomics. In this study, five distinct tumor molecular subgroups were identified on the basis of IHC, genomics, epigenetics, and transcriptomic analyses, which were reproducible in patient-derived xenograft models and thus allowed for in vivo drug sensitivity tests [139]. Beyond single gene analyses, mutational signatures, RNA-based gene expression profiling, immunophenotyping, and TMB determination have proven to be useful prognostic and predictive biomarkers of response to anticancer therapies, but whether they will lead to an increase in treatment opportunities is still unclear. The application of molecular profiling results in the clinical setting still faces several challenges**.** Current pitfalls and potential solutions are discussed below.

## Challenges and solutions for clinicians acting on molecular profiling results

Tremendous progress has been made in the field of precision medicine, with ever-increasing numbers of patients being tested and new biomarkers being developed leading to expanded therapeutic opportunities, but challenges remain. The results from target–drug matching initiatives have been disappointing to date, as most of these have matching rates of only 5–10% and the objective responses in genotype-matched patients have been modest (less than 20%; Table 2) [24–27, 96–98, 140–142]. There are multiple reasons for these low rates and the lack of objective responses in many genotype-matched patients. For instance, the disease may have progressed during the wait for sequencing results so that the patient is no longer fit for treatment, best-in-class therapeutic agents are not always available, poor response to a targeted agent may occur despite matching, there may be intratumoral heterogeneity, the treatment may be targeting a non-driver or passenger mutation, and there may be difficulties in combining targeted agents because of toxicity [143]. The systematic charting of successful and unsuccessful molecular treatment indications is still in its early stages. Efforts at data collection and sharing in order to provide evidence linking biomarkers to drugs and/or tumor types are required and need to be made public to guide treatment decision. For example, it has recently been shown that germline or somatic loss-of-function alterations in *BRCA1/2* are associated with tumorigenesis in only a few types of cancer, namely breast, ovarian, prostate, and pancreatic cancers, and that there is little benefit in treating other cancer types that harbor such mutations with PARP inhibitors [144].

**Table 2:** Selected molecular profiling initiatives and genotype matching to clinical trials

Group	Sample size	Platform	Tissue sample	Germline control	Patients enrolled in genotype-matched trials	ORR of patients matched to treatment based on genotype
MSKCC [27]	12,670	341–410 gene panels	FFPE	Yes	527/5009 (10.5%)	Not available
DFCI-HCC [28]	3727	275 gene panels	FFPE	No	16/50 (32%)	Not available
Lyon [140, 141]	2579	69 gene panels +aCGH	FFPE	Yes	182/2579 (7%)	13%
MDACC [26]	2000	11–50 gene panels	FFPE	No	83/2000 (4.2%)	Not available
Princess Margaret [25]	1640	23–48 gene panels	FFPE	Yes	92/1640 (5.6%)	19%
Goustave Roussy [24]	1035	30–75 gene panels + aCGH	Fresh biopsy	Yes	199/1035 (19.2%)	11%
Michigan [142]	556	WGS, WES, RNASeq	Fresh biopsy	Yes	3–11%	Not available

*aCGH* array comparative genomic hybridization, *DFCI-HCC* Dana Farber Cancer Institute-Harvard Cancer Center, *FFPE* formalin-fixed paraffin-embedded, *MDACC* MD Anderson Cancer Center, *MSKCC* Memorial Sloan Kettering Cancer Center, *ORR* objective response rate, *WES* whole exome sequencing, *WGS* whole genome sequencing

Multiple surveys have been undertaken to assess the utility of molecular profiling in patient care, and some of the issues identified that can limit access to potential treatment options include poor access to targeted agents, cost of targeted agents, and lack of clinical trial availability [143]. Limitations to the full implementation of precision medicine in routine clinical practice include the complexity of the molecular information generated, uncertainty surrounding the clinical utility of the information, lack of knowledge about this framework in general among healthcare professionals, and the economic costs of the tests. The number of genes that can be sequenced is very large, but not all of the genes will have practical application.

### Clinician education

Although large gene panel testing is being incorporated into clinical care, hurdles exist that may limit the applications of NGS by healthcare professionals to levels below their maximum potential. For instance, it has been shown that the results can be difficult to interpret, leading to the under- or over-interpretation of genomic information [145, 146]. The provision by clinical laboratories of NGS reports that include features such as a succinct summary of the genomic findings, written for a non-genetic specialist audience, will help with the decision-making process [147, 148]. A sample genomic report with information from a variety of resources can be seen in Table 3, which illustrates some of the challenges that face clinicians when interpreting annotations from different knowledgebases that are available for specific mutations. These challenges can include a lack of information regarding how a particular mutation or conflicting information from different knowledgebases should be interpreted. The development of easily accessible online genomic knowledge banks provides resources to aid data interpretation and clinical decision-making. Treating oncologists frequently cite perceived low levels of genetic knowledge or limited confidence in their ability to interpret genomic reports as reasons for lower utilization of genetic testing [151]. Several large institutions have created teams to centralize genomic interpretation and provide decision support. As an example, the Precision Oncology Decision Support (PODS) platform provides clinical decision support for oncologists at the MD Anderson Cancer Center. PODS offers a rapid and easily accessible means of obtaining scientifically curated information about the functional effects of genetic alterations, as well as information on genotype-matched therapeutics (including clinical trials) that are relevant to their patients [152, 153].

**Table 3:** Sample genomic report with several mutations of interest, which have varying degrees of actionability. Key information available through the CIViC [78, 149] and OncoKB [77, 150] databases for each variant is displayed in the table below the example report. The details of the CIViC variant evidence score [78, 149] and The OncoKB level of evidence system [77, 150] are available in the literature and on the relevant websites. Column 4 of the table displays the respective tier that the mutation falls into based on the AMP/ASCO classification for the interpretation of sequence variants in cancer [86]

**Mock molecular profiling report**			
**Patient identification** Name: Doe, Jane Subject number: XXXXXXXXX Diagnosis Tumor site/histology: head and neck/salivary Specimen(s) received 1. Consult slides—unstained—19:S1234 2. Consult slides—stained—19:S1234 Sample identifier: SEQ-01-1234		**Results** NGS panel results: positive Variant 1: MAP2K1 (NM_002755.3) c.171G>C (p.Lys57ASn) Percent variant: 42.5% Variant 2: TP53 (NM_000546.5) c. 469G>T (p.Val157Phe) Percent variant: 38.7% CNV 1:ERBB2 amplification Copy number: 177.0 Fusion 1: not detected	
**Methodology** Genomic DNA and RNA was extracted and analyzed using an NGS Panel that examines the coding regions (± 10 bp) of 500 genes using target enrichment hybrid capture followed by paired-end sequencing on the next sequencing platform. Variant calls are generated using a custom bioinformatics pipeline with alignment to genome build GRCh37/hg19. Minimum acceptable coverage for all reported genomic regions is > 200. The reportable range is 10–100% variant allele frequency. Test sensitivity is > 98% for detection of substitutions, small insertions or deletions, copy number changes, and RNA fusions. Large insertions or deletions, gene amplifications or loss, and some fusions may not be detected by this assay. Variants are interpreted only as somatic tumor variants because testing of DNA from germline tissue was not performed. Current methods may not detect all of the variants present in the genes tested. **Interpretation**			
Variant	CIViC database [78, 149]	OncoKB database [77, 150]	Standards and guidelines for the interpretation and reporting of sequence variants in cancer [86]
MAP 2 K1 (NM_002755.3) c.171G>C (p.Lys57ASn)	MAP2K1 is a dual-specificity kinase involved in the ERK pathway. Activating mutations have been seen in ovarian, melanoma, and lung cancers. Inhibitors of MEK genes have been shown to inhibit tumor growth. Evidence for K57N: 2 references This variant does not have a specific summary page Variant type: missense CIViC variant evidence score: 9.5 Drugs: selumetinib	Oncogenic: yes Mutation effect: gain-of-function Citations: 4 references Cancer type: low-grade serous ovarian cancer, melanoma, non-small cell lung cancer, histiocytosis Drugs: cobimetinib, trametinib Level of evidence: 3A	Tier IID—potential clinical significance Preclinical trials: few case reports without consensus • Rare in the head and neck (TCGA) • Gain-of-function variant
TP53 (NM_000546.5) c. 469G>T (p.Val157Phe)	TP53 mutations are universal across cancer types. Majority of mutations localize to the DNA binding domain Evidence for DNA binding mutation: 2 references Variant type: DNA binding site CIViC variant evidence score: 35 Drugs: none	Oncogenic: likely Mutation effect: likely loss-of-function Citations: 3 references Drugs: none Level of evidence: N/A	Tier IID—potential clinical significance Preclinical trials: few case reports without consensus • Non-functional variant (IARC TP53 database) • Seen in the head and neck (TCGA, COSMIC)
ERBB2 amplification	ERBB2/HER-2 is amplified or overexpressed in 20–30% of invasive breast cancers, commonly treated with HER-2 targeted therapy. Evidence for amplification: 60 references Variant type: transcript amplification CIViC variant evidence score: 822.5 Drugs: trastuzumab, pertuzumab, neratinib, lapatinib, TDM-1, afatinib, cetuximab	Oncogenic: yes Mutation effect: gain-of-function Citations: 6 references Cancer types: breast cancer, esophagogastric cancer, uterine serous carcinoma Drugs: lapatinib, trastuzumab, TDM-1, neratinib, pertuzumab Level of evidence: 2B	Tier IIC—potential clinical significance. FDA-approved therapy for different tumor site • ERBB2 inhibitors used in metastatic breast cancer • ERBB2 amplifications seen in head and neck (TCGA, COSMIC) Not approved for head and neck tumors
High TMB	No specific reference page	No specific reference page	No suitable category

With a longer vision, it will be important to educate future physicians and expose medical students to this growing area. Specialist medical oncology training should also involve a focus on precision medicine, and physicians should be encouraged to become actively involved in MTB. Continuing medical education training courses should provide a focus on precision medicine, and ensuring that there is adequate staffing in terms of genetic counselors, medical geneticists, and adequately trained physicians in this field is essential.

### Patient education

Another aspect of genomic healthcare involves the education of patients in order to facilitate their taking part in their own care. A significant proportion of the general public have difficulty in understanding health and specifically genetic information. There is a need for educational intervention research to help patients to understand test results and treatment options. As many patients are eager to undergo tumor sequencing, providers need to communicate its potential benefits as well as its risks and limitations clearly. Patients have high expectations for and interest in tumor sequencing, but they can be concerned about the complexity of the data, the potential for disappointment, and the loss of hope after testing (especially if no alterations are identified). The education of patients prior to testing is essential, but how best to execute this is unknown [154]. A helpful step in terms of improving patient engagement with their own care is the development of patient-friendly reports and patient-specific webpages, written in an accessible language in the knowledgebases being used by physicians. An important aspect of patient education in precision medicine surrounds the potential for the identification of secondary germline mutations and the potential to assess the patient’s preference for receiving incidental germline findings [155]. Studies indicate that up to 18% of patients undergoing tumor-normal sequencing have a pathogenic germline variant [156–158]. In addition, many providers may not feel qualified or have the time to have a discussion with their patients regarding secondary germline findings. Access to genetic counselors can be challenging in a community setting [159]. As a result, the development of virtual or telehealth genetic counseling support may be worth exploring. The COMmunication and Education in Tumor profiling (COMET) study, which is an ancillary study to NCI-MATCH, aims to examine whether educating patients who have cancer about genetic testing will increase their knowledge and reduce stress levels after receiving the results of tumor profiling [160].

### Increased trial opportunities

Traditional clinical trial designs may not offer an efficient investigation of precision medicine, and as a result, more flexible trial designs have been developed. Adaptive studies include inbuilt opportunities to modify one or more specified trial elements on the basis of intermediate data analysis. For instance, treatment arms may be opened or closed on the basis of provisional findings at pre-specified points, such as emerging evidence of response to treatment. This can increase efficiency by facilitating the selection of the dose, sparing patients from being exposed to ineffective doses, and reducing cost and duration of clinical development [161]. The evolution of the use of genomic results to guide treatment decisions in precision medicine has led to the increased use of “master protocols” or “platform trials,” in which multiple parallel studies operate under one over-arching protocol. These platforms, developed to allow the investigation of multiple target–treatment pairs in parallel, require close collaboration between industry, academic, and regulatory partners. As an example, the CAnadian Profiling and Targeted agent Utilization Trial (CAPTUR) [162] involves collaboration between pharmaceutical companies, Health Canada, the Canadian Clinical Trials Group (CCTG), and the individual health care facilities involved in running the study. Logistics can often be complicated, requiring multiple pharmaceutical companies to provide drugs to a trial. Basket trials test the effect of one drug on a single aberration in a variety of tumor types, greatly increasing the number of patients who are eligible to receive certain drugs. Conversely, in umbrella studies, patients with a specific cancer type are centrally screened and assigned to one of several molecularly defined subtrials investigating a matched targeted therapy. These trials are relatively flexible and allow for the addition of new treatment arms as new clinical data become available [163, 164].

A number of basket trials around the world are currently recruiting patients, some of which are highlighted in Table 4. These trials are testing commercially available targeted agents (and in some cases also investigational agents) in patients who have undergone tumor profiling. The results from three cohorts of one of these studies, the NCI-MATCH study, have been reported: patients with *ERRB2/HER2* amplification [168], *FGFR* alterations [169], or *PIK3CA* [170] mutations were treated with T-DM1, AZD4547, or taselisib, respectively. Unfortunately, objective response rates were low in all three groups, ranging from 0 to 9.5%. Reasons that might account for these low responses include that the patients were heavily pre-treated or the presence of co-occurring mutations. Numerous umbrella protocol studies are ongoing, one example being the Adjuvant Lung Cancer Enrichment Marker Identification and Sequencing Trials (ALCHEMIST), which is investigating the use of targeted therapy in patients with resectable adenocarcinoma of the lung with *EGFR* mutation or *ALK* translocation after completion of standard therapy [171].

**Table 4:** Selected examples of ongoing large genotype–drug matching PCM trials

Name	Site	Sample size	Mutations matched	Targeted drugs used
NCI-MATCH [165]	National Cancer Institute (NCI)	6452	EGFR/HER2-activating mutation	Afatinib
			MET, ALK, ROS1	Crizotinib
			EGFR T790M or other activating mutation	Osimertinib
			BRAF V600E/R/K/D, BRAF fusion, non-BRAF V600 mutations	Dabrafenib+trametinib
			NF1, GNAQ, GNA11	Trametinib
			PIK3CA	Taselisib
			HER-2 amplification	Trastuzumab+pertuzumab
			FGFR mutation or fusion	Erdafitinib
			mTOR, TSC1, TSC2	Sapanisertib
			PTEN mutation	GSK2636771 (PI3K beta inhibitor)
			HER-2 amplification	Trastuzumab, emtansine
			SMO, PTCH1	Vismodegib
			NF2 inactivating mutation	Defactinib
			cKIT mutation	Sunitinib
			FGFR1, FGFR2, FGFR3 mutation	AZD4547 (FGFR inhibitor)
			Certain DDR2 mutations	Dasatinib
			AKT mutation	Capivasertib
			NRAS mutations	Binimetinib
			CDK4, CDK6	Palbociclib
			Mismatch repair deficiency	Nivolumab
			NTRK1, NTRK2, NTRK3 fusions	Larotrectinib
			PIK3CA, PTEN mutations	Copanlisib
			BRCA1, BRCA2 mutation	Adavosertib
			AKT mutation	Ipatasertib
			BRAF non-V600 mutation or BRAF fusion	Ulixertinib
TAPUR [166]	American Society of Clinical Oncology (ASCO)	3123	ALK, ROS1, MET	Crizotinib
			CDKN2A, CDK4, CDK6	Palbociclib
			CSF1R, PDGFR, VEGFR	Sunitinib
			mTOR, TSC	Temsirolimus
			ERBB2	Trastuzumab+pertuzumab
			BRAFV600E/D/K/R	Vemurafenib+cobimetinib
			NRAS, KRAS, NRAF	Cetuximab
			BCR-ABL, SRC, KIT, PDGFRB, EPHA2, FYN, LCK, YES1	Dasatinib
			RET, VEGFR1/2/3, KIT, PDGFRB, RAF-1, BRAF	Regorafenib
			BRCA1, BRCA2, ATM	Olaparib
			POLE, POLD1, high mutational load	Pembrolizumab
			MSI-high, high mutational load and others	Nivolumab+ipilimumab
CAPTUR [162]	Canadian Cancer Trials Group (CCTG)	720	VEGFR1, VEGFR2, VEGFR2	Axitinib
			BCR-ABL, SRC	Bosutinib
			ALK, ROS1, MET	Crizotinib
			KIT, PDGRFA, PDGFRB, ABL1	Dasatinib
			EGFR	Erlotinib
			High mutation burden, POLE, POLD1	Nivolumab+ipilimumab
			BRCA1, BRCA2, mutations in HRD	Olaparib
			CDKN2A, CDK4, CDK6, CCND1	Palbociclib
			CSF1R, PDGFRA, PDGFRB, VEGFR1, VEGFR2, VEGFR3, KIT, FLT3, RET, FGFR1, FGFR2, FGFR3, VHL	Sunitinib
			AKT1, AKT2, AKT3, FBXW7, FLCN, mTOR, NF1, NF2, NTRK3, PIK3CA, PIK3R1, PTEN, RHEB, STKII, TSC1, TSC2	Temsirolimus
			ERBB2	Trastuzumab+pertuzumab
			BRAFV600	Vemurafenib+cobimetinib
			PTCH1, SMO	Vismodegib
DRUP [167]	Netherlands Cancer Institute	400	KRAS, BRAF, NRAS wild type	Panitimumab
			BRCA1, BRCA2, ATM	Olaparib
			BRAF	Dabrafenib
			Molecular profile that can potentially be targeted by nilotinib	Nilotinib
			Molecular profile that can potentially be targeted by trametinib	Trametinib
			Molecular profile that can potentially be targeted by erlotinib	Erlotinib
			HER-2 overexpression, amplification or mutated	Trastuzumab+pertuzumab
			BRAF mutated tumors	Vemurafenib+cobimetinib
			Molecular profile that can potentially be targeted by vismodegib	Vismodegib
			Molecular profile that can potentially be targeted by regorafenib	Regorafenib
			Molecular profile that can potentially be targeted by nivolumab	Nivolumab

The Drug Rediscovery Protocol (DRUP) is an ongoing Dutch adaptive precision oncology trial that facilitates the use of approved drugs beyond their approved indication in rare cancer subgroups. Initial results from the two subgroups that have completed data accrual have been published. The first was in agnostic microsatellite instable (MSI) tumors treated with nivolumab. The European Medicines Agency has not yet approved the use of checkpoint inhibitors in this setting, but on the basis of the positive results from DRUP, the Dutch regulatory agency has now approved the use of these drugs in this indication. The second cohort in microsatellite stable colorectal cancer with TMB between 11 and 22 mutations per megabase showed limited clinical benefit, leading to the closure of the arm [39, 167, 172].

By and large, current platform trials are exploring targeted agents given as monotherapies. Genomic alterations do not always lead to oncogenic pathway activation or addiction, whereby certain tumor cells become dependent on a single activated oncogenic protein or pathway. As a result, targeting of multiple driver and/or resistance pathways using combinatorial approaches may be required for optimal antitumor activity [173]. The iPREDICT study is matching patients to combination therapies on the basis of genomic results interpreted by the study’s MTB. Initial results from 73 patients treated with bespoke personalized therapy showed that 30% of the patients achieved disease control. As many of the combinations had not been tested for safety, patients were initially started on low doses of the drugs, which were increased to a level that was well tolerated by each patient [174].

### Trial matching resources

Numerous components are involved in automating the matching of patients to clinical trials, including creating a database and then establishing a method to search the database to match patients to trials. In addition, the maintenance of up-to-date comprehensive databases is necessary for the automation of patient–trial matching [175]. The Phase One Spot Tracker (POST) is an online secure database that has been set up in Princess Margaret Cancer Centre. This database contains key trial eligibility criteria and can be used to help to identify patients for trials on the basis of their tumor type and molecular signature (https://uhnddp.ca) [176]. Furthermore, Matchminer, developed by the Dana Farber Cancer Institute, is an example of an open-source computational platform that matches patient-specific genomic events to clinical trials and makes the results available to trial investigators and clinicians via a web-based platform [177]. The use of artificial intelligence to improve the matching of patients to trials has also been investigated. For instance, the Watson for Clinical Trial Matching cognitive system uses natural language processing to derive patient and tumor attributes from electronic health records and to match the data to clinical trial eligibility criteria. This platform has been found to increase clinical trial enrollment of breast cancer patients [178].

## Conclusions

The implementation of precision medicine through molecular profiling technologies has increasingly been integrated with standard clinicopathological evaluations to enhance diagnosis, prognostication, and prediction of clinical outcomes. Although there have been clear successes in the era of molecular characterization, the utility of NGS and other omics-based tests remains unproven on many fronts. A vision for the future of precision medicine will integrate comprehensive multi-omic tumor characterization, dynamic monitoring of liquid biopsy samples, annotation that is automated through advancements in artificial intelligence but guided by experts’ clinical input, the enrollment of patients into innovative clinical trials that not only test molecular profile–drug matching but also investigate the utility of different drug-assignment algorithms [179], and the real-time addition of information from each case to global knowledgebases to enhance precision cancer medicine learning. The path forward in precision medicine will require not only extension beyond genomics from a technical viewpoint, but also the education and engagement of end-users such as clinicians and patients, the increase of access to genotype–drug matching through adaptive and other innovative clinical trial designs, and the promotion of data sharing to maximize knowledge gain.

## Supplementary information

**Additional file 1.** Advantages and disadvantages of NGS-based approaches.

## Abbreviations

CNV: Copy number variations
CTC: Circulating tumor cell
ctDNA: Circulating tumor DNA
FISH: Fluorescence in situ hybridization
IHC: Immunohistochemistry
IO: Immuno-oncology
miRNA: MicroRNA
MTB: Molecular tumor board
NGS: Next-generation sequencing
NSCLC: Non-small cell lung cancer
SNV: Single nucleotide variants
TCGA: The Cancer Genome Atlas
TMB: Tumor mutation burden
WES: Whole exome sequencing
WGS: Whole genome sequencing
