# Supplementary material for: Molecular profiling for precision cancer therapies
Source: Genome Med. 2020 Jan 14;12:8. doi: 10.1186/s13073-019-0703-1 (PMC6961404; doi:10.1186/s13073-019-0703-1)
Supplement: Supplementary file 1 — Additional file 1. Advantages and disadvantages of NGS-based approaches. [file 13073_2019_703_MOESM1_ESM.docx]

| **NGS Assay** | **Advantages** | **Disadvantages** |
| --- | --- | --- |
| **Targeted panels** | - Customization of gene sets and regions of interest based on clinical significance - Higher depth of coverage of known actionable variants - Easier interpretation of results - Faster and cheaper (depending on the panel size) - Assessment of tumor mutational burden (feasible in large panels) | - No information beyond genes included in the panel - Limited evaluation of unknown somatic variants |
| **Whole-exome sequencing** | - Detection of novel somatic variants - Assessment of tumor mutational burden - MSI status can be determined - Less expensive and time consuming than WGS | - Requires enrichment of areas of interest and bioinformatic analysis (variation across centres leading to bias) - Limited assessment of structural variants - Increased detection of variants of unknown significance - Difficult interpretation of results - Higher sample requirements |
| **Whole-genome sequencing** | - Highest sensitivity to detect structural variants (CNV and regions of LOH) - MSI status can be determined - Most comprehensive assessment (coverage of non-coding regions more) - Does not require enrichment | - Lower depth of coverage: less sensitivity to detect known variants. - Difficult interpretation of results - Most expensive and time consuming - Increased chance of incidental findings (e.g. pathogenic germline variants) - Higher sample requirements |

**Additional file 1**

**Supplementary Table 1:** Advantages and disadvantages of NGS-based approaches.

Abbreviations: MSI= microsatellite instability; CNV = copy number variants; LOH: loss of heterozygosity
